# *Rhodotorula mucilaginosa* Supplementation Could Significantly Affect the Growth Performance, Digestive Enzyme Activity, Antioxidant Capacity, Immune Function, and Intestinal Health in Red Claw Crayfish (*Cherax quadricarinatus*)

**DOI:** 10.3390/biology14091164

**Published:** 2025-09-01

**Authors:** Qin Zhang, Yuguan Liang, Jiqing Li, Luoqing Li, Liuqing Meng, Qinghui Zeng, Dapeng Wang, Rui Wang, Tong Tong, Yongqiang Liu, Huizan Yang

**Affiliations:** 1 Guangxi Key Laboratory for Polysaccharide Materials and Modifications, Guangxi Marine Microbial Resources Industrialization Engineering Technology Research Center, School of Marine Sciences and Biotechnology, Guangxi Minzu University, 158 University Road, Nanning 530008, China; zhangqin@gxmzu.edu.cn (Q.Z.); liangyuguan@stu.gxmzu.edu.cn (Y.L.); lijiqing@stu.gxmzu.edu.cn (J.L.); liluoqing@stu.gxmzu.edu.cn (L.L.); mengliuqing@stu.gxmzu.edu.cn (L.M.); zengqinghui@stu.gxmzu.edu.cn (Q.Z.); tongtong@gxmzu.edu.cn (T.T.); 2 Guangxi Key Laboratory for Aquatic Genetic Breeding and Healthy Aquaculture, Guangxi Academy of Fishery Science, 8 Qingshan Road, Nanning 530021, China; oucwdp@163.com (D.W.); raywongxx@163.com (R.W.)

**Keywords:** red claw crayfish, *Rhodotorula mucilaginosa*, growth performance, immuno-antioxidant capacity, intestinal health

## Abstract

**Simple Summary:**

Red claw crayfish (*Cherax quadricarinatus*) is an important aquatic species, but its growth and health can be affected by poor water conditions and diseases in intensive farming. To address this, we studied whether adding *Rhodotorula mucilaginosa* to their feed could help. We fed crayfish different amounts of this yeast (0, 0.1, 1.0, and 10.0 g/kg of feed) for 56 days and observed their growth, digestion, immunity, and gut health. The results showed that adding the yeast improved the crayfish’s growth. It also helped them digest food better by increasing the activity of digestive enzymes. The yeast boosted their ability to fight off diseases by enhancing immune-related functions and reduced harmful bacteria in their guts while increasing beneficial ones. The best effect was seen when adding 1.0 g/kg of yeast to the feed. These findings can help farmers raise healthier crayfish more efficiently, supporting sustainable aquaculture and providing a safer food source for people.

**Abstract:**

This study investigated the effects of dietary *Rhodotorula mucilaginosa* supplementation with different concentrations (0.0 g/kg, 0.1 g/kg, 1.0 g/kg, 10.0 g/kg) on red claw crayfish (*Cherax quadricarinatus*). Four groups were established: control group (CK, 0.0 g/kg), low-dose group (HL, 0.1 g/kg), medium-dose group (HM, 1.0 g/kg), and high-dose group (HH, 10.0 g/kg). The feeding trial lasted for 56 days. The results showed that, compared with the control group, all supplementation groups exhibited significantly reduced feed conversion ratios (*p* < 0.05). The HM and HH groups demonstrated significant increases in body length growth rate, specific growth rate, weight gain rate, hepatosomatic index, and survival rate (*p* < 0.05). All supplemented groups showed significantly enhanced trypsin and lipase activities in intestines and trypsin activity in the hepatopancreas (*p* < 0.05). The HM and HH groups exhibited elevated α-amylase activity in the hepatopancreas (*p* < 0.05). Compared with the control group, marine red yeast supplementation reduced colonization of potential pathogens while increasing probiotic abundance, effectively improving intestinal microbiota structure. The HM group significantly improved intestinal villus length, width, and muscular thickness (*p* < 0.05). All supplemented groups showed considerable upregulation of hepatopancreatic genes related to immunity (heat shock protein 70, down syndrome cell adhesion molecule, crustacean antibacterial peptide, serine proteinase inhibitors, crustacean hyperglycemic hormone, anti-lipopolysaccharide factor, lysozyme, and alkaline phosphatase) and antioxidant defense (superoxide dismutase, glutathione peroxidase, glutathione, and catalase) (*p* < 0.05). These findings indicate that *R. mucilaginosa* can significantly enhance digestive enzyme activity, maintain intestinal health, improve antioxidant and immune-related gene expression, and promote growth performance in red claw crayfish, with the HM group (1.0 g/kg *R. mucilaginosa*) showing optimal promotion effects.

## 1. Introduction

Red claw crayfish (*Cherax quadricarinatus*), commonly known as the Australian freshwater crayfish, is an omnivorous bottom-dwelling species. Due to its large size and hard exoskeleton, it is less vulnerable to predation by fish [1]. It has received widespread attention for its high reproductive capacity and rapid growth. Under suitable cultural conditions, it can reach marketable size in only 6–9 months [2], which, together with its tasty meat and ability to adapt to a variety of environmental conditions, makes it one of the most economically valuable species in current aquaculture in China [3]. Global red claw crayfish production has increased significantly from 150,000 metric tons in the late 1990s to over 1,000,000 metric tons in 2017, with sales in the Chinese market reaching USD 2 billion that year [4]. As aquaculture continues to advance toward high-density intensification, the aquatic environment has undergone significant degradation, negatively impacting the immune systems of aquatic organisms and contributing to the onset of various diseases. This impact is particularly pronounced in relation to growth performance and intestinal health [5]. To prevent disease outbreaks in red claw crayfish, farmers commonly rely on antibiotics as a primary method for disease management. However, the excessive use of antibiotics not only contributes to the development of drug-resistant pathogens but also results in environmental pollution and ecological imbalance [6]. Studies have shown that probiotics provide multiple health benefits to the host, including improved intestinal health and a balanced gut microbiota. These benefits are essential for digestion, nutrient absorption, immunity, and disease resistance. Probiotics exert their effects through mechanisms such as competitive inhibition of pathogenic microorganisms, enhancement of immune and antioxidant capacity, and improved nutrient digestion and absorption, ultimately supporting host growth [7]. Additionally, probiotics offer the advantages of high safety, environmental friendliness, and no side effects [8], making them a more effective feed additive than antibiotics for maintaining aquatic animal health.

The marine red yeast (*Rhodotorula mucilaginosa*), widely distributed in soils, aquatic environments, and plant surfaces [9], is recognized for its ability to synthesize multiple carotenoids, including β-carotene and γ-carotene, which confer distinctive orange–red to pink pigmentation to its colonies. This species exhibits robust growth capabilities, efficiently utilizing diverse carbon sources while demonstrating notable environmental stress tolerance. Additionally, it serves as a rich source of bioactive compounds, particularly polysaccharides, such as β-glucan and mannan oligosaccharides (MOS), along with nucleotides [10]. Studies have demonstrated that β-glucan and MOS enhance immune responses by improving resistance against infectious pathogens. The immunomodulatory efficacy of β-glucan is primarily mediated through two mechanisms: elevation of antibody titers and activation of macrophage phagocytic activity, thereby reinforcing host defense systems [11]. As a critical cell wall component, MOS supplementation in animal feed has been evidenced by multiple studies to significantly improve growth performance and feed utilization efficiency while concurrently enhancing antibody levels, lysozyme activity, and alternative complement pathway activity [12]. In recent years, *R. mucilaginosa* has been widely used in aquaculture: the addition of appropriate amounts of *R. mucilaginosa* to the diets of gibel carp (*Carassius auratus gibelio*) [13] and Nile tilapia (*Oreochromis niloticus*) enhances the non-specific immune response, upregulates expression of immune-related genes, and strengthens disease resistance [14], and the feed addition of marine red yeast (*R. mucilaginosa*) can improve the growth performance, survival rate, and antioxidant activity of golden pomfret (*Trachintus ovatus*). In addition, yeast can be used as an immunostimulant for aquatic animals to enhance the immune response and disease resistance of farm animals against *Aeromonas hydrophila* [13] and *Vibrio harveyi* [15]. As a multifunctional feed additive, *R. mucilaginosa* is rich in various nutrients and bioactive substances that not only promote the growth performance of aquatic animals but also play an important role in regulating the intestinal flora of aquatic animals and maintaining intestinal health [16].

As a freshwater economic crustacean, the red crayfish exhibits notable physiological and metabolic differences from fish, such as in molting cycle and intestinal flora structure, and the effects of yeast additives may be species specific. Most existing studies have primarily focused on the isolated impacts of yeast on growth performance or immune response, with a lack of systematic evaluations on digestive enzyme activity, antioxidant capacity, intestinal health, and immune-related gene expression. Therefore, this study aimed to evaluate the effects of *R. mucilaginosa* on growth performance, digestive enzyme activities, antioxidants, immunity, and intestinal health of red claw crayfish by supplementing different levels of *R. mucilaginosa* in basal feeds fed to them. The results of this study will provide a scientific basis for the development and application of red yeast in aquafeeds.

## 2. Materials and Methods

### 2.1. Experimental Diets

The base feed of this study was crayfish compound feed produced by Guangdong Hengxing Feed Industry Co., Ltd. (Guangzhou, China). The dry matter content of the basic feed was 92.56%, crude fat content was 7.84%, crude protein content was 35.20%, and crude ash content was 7.76%. The *R. mucilaginosa* used in the experiment was a laboratory-preserved strain (the number of live bacteria was >10^10^ CFU/mL) and provided by Guangzhou Xinhaili Biotechnology Co., Ltd. (Zhanjiang, China). The nutritional composition of *R. mucilaginosa*, expressed on a wet weight basis, comprised 81.17% moisture, 9.27% crude protein, 4.45% crude fat, 4.20% total triglycerides, and 1.30% β-glucan. Additionally, it contained 1.40 mg/kg of β-carotene, 1.00 mg/kg of astaxanthin, and 172.00 mg/kg of vitamin E.

According to the previous research experience [17], four different contents of *R. mucilaginosa* feeds were made by evenly spraying the *R. mucilaginosa* liquid on the surface of the basic feeds: 0 CFU/g (CK group), 1 × 10^6^ CFU/g (HL group), 1 × 10^7^ CFU/g (HM group), and 1 × 10^8^ CFU/g (HH group), respectively.

The original solution of *R. mucilaginosa*, with a concentration of 10^10^ CFU/mL, was serially diluted with sterile physiological saline to achieve a concentration consistent with the required feed supplementation level (The HL group was diluted to 10^6^ CFU/mL, the HM group was diluted to 10^7^ CFU/mL, and the HH group was diluted to 10^8^ CFU/mL) to prevent local aggregation caused by high-concentration bacterial solutions. The diluted bacterial suspension was then uniformly sprayed onto the surface of the basal feed at a rate of 10 mL/kg of feed using a laboratory-specific sprayer. During this process, the feed tray was agitated at a speed of 50 revolutions per minute to ensure even coverage of the bacterial solution on the feed particles. Following the preparation of each batch of feed, five samples (1 g each) were randomly collected from different locations within the batch. The number of viable bacteria in each sample was determined using the plate counting method [18]. The results indicated that the coefficient of variation (CV) of viable bacterial counts across the samples was less than 5%, demonstrating a uniform distribution of yeast in the feed with no significant local concentration differences. After the experimental feeds were made, they were protected from light, dried, and stored in the refrigerator at −20 °C. To ensure the number of viable bacteria of *R. mucilaginosa*, the experimental feeds were made every 7 days.

To monitor the reduction in viability of live bacteria during feed storage, samples were randomly collected from the same batch of feed at various time points during storage, and the survival rate of the bacteria was calculated. The number of viable *R. mucilaginosa* in the prepared feeds was determined and analyzed by the plate counting method [18]: Weigh 1 g of feed sample, place it into 9 mL of sterilized saline, and prepare the stock solution by vortex mixing uniformly; dilute from the stock solution to 10^−3^ to 10^−8^; take 0.1 mL of each dilution, spread it evenly on sterilized nutrient agar (NA) medium, respectively, and make three replicates for each dilution concentration; incubate anaerobically at 35 °C for 48 h; count the number of colonies in the plates containing marine *R. mucilaginosa*; after colony counting, randomly select colonies for further identification and isolate the *R. mucilaginosa* in the feed. The number of colonies was counted; after colony counting, colonies were randomly selected for further identification and isolated to *R. mucilaginosa*. The number of *R. mucilaginosa* in the plate was calculated by the following formula:

*R. mucilaginosa* viable count (CFU/g) = B × (C/10) × f (1)

Note: In the above equation, B is the total number of plate colonies on NA agar, C is the number of *R. mucilaginosa* colonies identified from 10 colonies, and f is the number of dilutions.

It was found that no *R. mucilaginosa* was detected in the CK group based on NA plate counts, and the live *R. mucilaginosa* counts in the feed of the HL, HM, and HH groups were 0.89 ± 0.13 × 10^6^, 0.87 ± 0.16 × 10^7^, and 0.92 ± 0.11 × 10^8^ CFU/g, respectively.

### 2.2. Experimental Animals and Culture

The red claw crayfish used in the experiment came from the South Breeding Base of Guangxi Academy of Marine Sciences (Nanning, China), and robust red claw crayfish fry with consistent specifications were selected, with an initial body weight of 0.13 ± 0.06 g and an initial body length of 0.58 ± 0.02 cm. The experimental design in this study was approved by the Biomedical Ethics Committee of Guangxi Minzu University, Nanning, China (Approval No. GXMZU-2023–018). Before the experimental culture, all the red claw crayfish fry were temporarily reared for 7 days in an outdoor system to allow them to recover from transportation stress and acclimate to the experimental rearing conditions, including water temperature, light cycle, and basal diet, and juvenile crayfish with consistent body size, no limb defects, a healthy appearance, and energetic and in the intermolt phase were taken, and then, the subsequent culture experiment was carried out. Subsequent culture experiments were conducted. The size of the culture tanks used in the experiments was 1 × 2 × 1 m, the water depth was 0.7 m, and 16 PVC pipes were arranged in the tanks as a hiding place. Inside each tank, air stones were used to increase oxygen, a certain amount of water plants were placed, and the water temperature was maintained at a suitable range of 26~27 °C, the pH value was in the range of 7.6~7.8, and natural light was used to create a natural ecological aquaculture environment.

Four groups of juveniles with uniform specifications were established: control group (CK, 0.0 g/kg), low-dose group (HL, 0.1 g/kg), medium-dose group (HM, 1.0 g/kg), and high-dose group (HH, 10.0 g/kg), three replicates in each group, a total of 12 aquaculture tanks, and 50 crayfish fries in each aquaculture tank, totaling 600 crayfish. During the formal culture period, the growth status and the number of deaths of red claw crayfish in each group were observed and recorded before each feeding. The daily feeding amount was about 5% of the crayfish’s body weight and was adjusted according to the actual feeding situation of the red claw crayfish. Feeding was carried out twice a day, at 8:30 and 18:30, and the ratio of morning and evening feeding was 3:7. After feeding in the morning, the red claw crayfish were cleaned of their excreta, and the water in the culture tank was replaced, with one-third of the total amount of water in the tank being replaced each time. The culture trial lasted for 56 days.

### 2.3. Sample Collection and Processing

At the end of the culture trial (56 d), all red claw crayfish were subjected to a 24 h fast. The weight and length of the red claw crayfish were determined by the group using electronic scales (accuracy ± 0.01 g) and measuring tape, respectively. An anticoagulant was prepared, which consisted of glucose 20.5 g/L, sodium citrate 8 g/L, sodium chloride 4.2 g/L; and the pH was adjusted to 7.5 and then pre-cooled and set aside. Twelve red claw crayfish were randomly selected from each replicate group, rinsed with sterile saline, and placed on ice (−10–0 °C) for anesthesia. Using a 1 mL disposable syringe to aspirate 300 μL of pre-cooled anticoagulant, hemolymph was drawn from the hemocoel at the base of the first abdominal segment of the red claw crayfish. After removing the needle, the hemolymph was slowly injected into a 1.5 mL Eppendorf tube and centrifuged at 4 °C and 1000 *g* for 10 min, and then, the supernatant was transferred to a new Eppendorf tube and stored in a −80 °C refrigerator.

Subsequently, the red claw crayfish were transferred to an ultra-clean bench and dissected under aseptic conditions, and the intestines were removed and placed in new Eppendorf tubes. The intestines of 5 red claw crayfish were frozen in liquid nitrogen and used for intestinal microbiological analysis, while the intestines of the other 5 red claw crayfish were frozen in liquid nitrogen, homogenized, and centrifuged for 10 min at 4 °C and 1000× *g*, and the supernatant was used for the subsequent determination. At the same time, the intestines of two red claw crayfish were collected to make intestinal sections. The hepatopancreas was removed, weighed, and then frozen in liquid nitrogen for subsequent determination. Muscle tissues from 5 red claw crayfish were mixed into one tube. All samples were stored at −80 °C in a refrigerator for subsequent analysis.

### 2.4. Growth Parameter Determination

Weight growth rate (WGR), survival growth rate (SGR), body length growth rate (BLG), hepatosomatic index (HSI), feed conversion rate (FCR), and survival rate (SR) were calculated using the following formulas:

(2) $$\mathrm{WGR}(\%)\;=\;\frac{W_{1}-W_{0}}{W_{0}}\;\times \;100\%$$

(3) $$\mathrm{SGR}(\%/d)=\frac{\ln W_{1}\;-{\;\ln W}_{0}}{T}\;\times \;100\%$$

(4) $$\mathrm{BLG}(\%)=\frac{L_{1}-L_{0}}{L_{0}}\;\times \;100\%$$

(5) $$\mathrm{HSI}(\%)=\frac{W_{h}}{W_{1}}\;\times \;100\%\;$$

(6) $$\mathrm{FCR}=\frac{W_{S}}{W_{1}-{\;W}_{0}}\times 100\%$$

(7) $$\mathrm{SR}(\%)=\frac{\mathrm{final}\;\mathrm{amount}\;\mathrm{of}\;\mathrm{crayfish}\;}{\mathrm{initial}\;\mathrm{amount}\;\mathrm{of}\;\mathrm{crayfish}}\times 100\%$$

In the above equations, W_1_ is the final weight (g), W_0_ is the initial weight (g), W_h_ is the liver weight (g), W_S_ is the total amount of diet weight (g), T is the number of days of feeding, L_0_ is the initial body length (cm), and L_1_ is the final body length (cm).

### 2.5. Determination of Enzyme Activities

The method of Zhang et al. [19] was applied to determine the digestive enzyme activities in the intestines and hepatopancreas of red claw crayfish.

The samples of intestines and hepatopancreas were thawed by removing them from the −80 °C refrigerator and then rinsed with saline and dried with filter paper. A total of 1 g of intestines and hepatopancreas was weighed accurately and put into the prepared 50 mL sterile and enzyme-free centrifuge tubes, 9 times the volume of saline was added into the centrifuge tubes, the centrifuge tubes were placed on ice for mechanical homogenization, the homogenates were centrifuged for 10 min at 4 °C under the condition of 1000× *g*, and then, the supernatants were taken, the homogenates were diluted with saline into a concentration of 1%, and then, the enzyme activities were detected.

Trypsin activity was determined at 37 °C, where 1 unit (U/mg prot) corresponds to the liberation of 1 μg tyrosine equivalent per milligram of tissue protein per minute. α-amylase activity was assessed via the starch–iodine colorimetric assay, with one unit defined as the quantity of enzyme hydrolyzing 10 mg starch in 30 min at 37 °C per milligram of tissue protein (U/mg prot). Lipase activity was measured by the microplate assay and expressed in units per gram of protein (U/g prot). All assay kits employed in this study were obtained from the Nanjing Jiancheng Bioengineering Institute (Nanjing, China), with all experimental procedures strictly adhering to the manufacturer’s detailed protocols.

### 2.6. Determination of Gene Expression

The real-time quantitative polymerase chain reaction (RT-qPCR) analysis method of Liu et al. [20] was applied to determine the relative expression levels of genes in the hepatopancreas of red claw crayfish.

The primers of superoxide dismutase (*sod*), heat shock protein 70 (*hsp70*), Down syndrome cell adhesion molecule (*dscam*), crustacean antibacterial peptide (*crustins*), glutathione peroxidase (*gpx*), glutathione (*gsh*), catalase (*cat*), serine proteinase inhibitors (*spi*), crustacean hyperglycemic hormone (*chh*), anti-lipopolysaccharide factor (*alf*), lysozyme (*lzm*), alkaline phosphatase (*alp*), and *β-actin* were designed and synthesized by Shanghai Sangon Bioengineering Technology Co., Ltd., located in Shanghai, China. Detailed information about the primers can be found in Table 1. *β-actin* was used as a non-regulatory internal reference gene. Real-time quantitative PCR (RT-qPCR) was performed using the TB Green^®^ Premix Ex Taq™ II (Tli RNaseH Plus) kit (TaKaRa Bio Inc., Beijing, China) on a LongGene Q2000B Real-Time PCR System (Roche, Basel, Switzerland). Analysis of the primer dissociation curves showed that all primers exhibited single peaks, indicating specific amplification, and thus could be used for subsequent experimental analysis.

The 2^−∆∆CT^ method was applied to calculate the relative expression levels of *sod*, *gpx*, *gsh*, *cat*, *hsp70*, *alf*, *chh*, *alp*, *dscam*, *crustins*, *lzm*, and *spi* genes in the hepatopancreas of red claw crayfish [21].

### 2.7. Intestinal Histology and Microbiota Profiling

Two live red claw crayfish per culture tank (biological replicate), with 3 tanks per treatment group, were sampled per experimental replicate group. After dissection, intestinal segments approximately 0.5 cm in length were excised and fixed in 4% neutral-buffered formalin for 12 h. The tissues were then dehydrated through an ethanol gradient to remove moisture, followed by clearing in xylene. The cleared intestinal samples were embedded in paraffin and sectioned. Sections were stained with hematoxylin and eosin (H&E). Intestinal histological features, including villus length (VL), villus width (VW), and mucosal thickness (MT), were observed and recorded using an optical microscope [22].

Five live red claw crayfish per culture tank (biological replicate), with 3 tanks per treatment group, were sampled as replicate groups for intestinal microbiota analysis. Small sections from both the anterior and posterior ends of the intestinal tract were excised using sterile forceps and scissors, and the midgut section was retained. Each midgut sample was transferred to a 2 mL sterile cryovial for sequencing (sample size > 0.1 g).

### 2.8. Bacterial DNA Extraction and High-Throughput Sequencing

Total DNA was extracted from intestinal tissues and luminal contents of red claw crayfish using the HiPure Stool DNA Kit (Magen Biotech, Guangzhou, China). The V3-V4 region of the bacterial 16S rRNA gene was amplified via targeted PCR with the specific primers 341F (5′-CCTACGGGNGGCWGCAG-3′) and 806R (5′-GGACTACHVGGGTATCTAAT-3′). The PCR amplification was performed in a total reaction volume of 50 μL containing the following: 5 μL of 10× KOD Buffer, 5 μL of 2 mmol/L dNTPs, 1.5 μL of 10 μmol/L forward primer, 1.5 μL of 10 μmol/L reverse primer, 1 μL of KOD Polymerase, 3 μL of 25 mmol/L MgSO_4_, 100 ng of template DNA, and ddH_2_O to adjust the final volume to 50 μL. The PCR protocol consists of the following steps: Initial denaturation at 94 °C for 2 min to completely unwind DNA strands, followed by the amplification phase (30 cycles) comprising three stages per cycle (10 s high-temperature denaturation at 98 °C for strand separation, 30 s annealing at 62 °C to facilitate primer–template specific binding, and 60 s extension at 68 °C for nascent strand synthesis). After amplification, a final 5 min extension at 68 °C ensures product integrity. Post-PCR analysis involves product verification through 2% agarose gel electrophoresis. Subsequently, the target DNA bands were purified using the AxyPrep DNA Gel Extraction Kit (AxyPrep; Axygen Scientific, Inc., Union City, CA, USA) and quantitatively analyzed with the ABI StepOnePlus Real-Time PCR System (Applied Biosystems™ StepOnePlus™ Real-Time PCR System, Thermo Fisher Scientific, Foster City, CA, USA). Finally, samples were submitted to Guangzhou Gidio Biotechnology Co., Ltd. (Guangzhou, China) for sequencing on the MiSeq PE250 high-throughput sequencing platform (Illumina, San Diego, CA, USA). All procedures were conducted in strict accordance with the manufacturer’s protocols.

### 2.9. Bioinformatics Analysis

The high-throughput sequencing data of intestinal microbiota from red claw crayfish were analyzed using Omicsmart, a real-time interactive online data analysis platform provided by Guangzhou Gidio Biotechnology Co., Ltd. (Guangzhou, China). Raw sequences from all samples underwent quality control and assembly. Operational taxonomic unit (OTU) clustering analysis was performed by grouping high-quality reads into OTUs at a 97% similarity threshold, with low-abundance OTUs (<3 reads) filtered as sequencing noise. The absolute tag abundance and relative distribution of each OTU across samples were calculated. Representative sequences of OTUs were taxonomically classified using the RDP classifier (Ribosomal Database Project classifier). Alpha diversity analysis was conducted, followed by Kruskal–Wallis rank-sum tests to assess significant differences among the four intestinal microbial groups. Bacterial community structures and relative abundances were analyzed at the phylum and genus levels. Finally, functional prediction was performed with Tax4Fun to infer potential KEGG metabolic pathways, with statistical analysis of KEGG orthology (KO) abundances characterizing the functional profiles of intestinal microbiota across sample groups.

### 2.10. Data Statistics and Analysis

The experimental data were recorded and preliminarily organized using Microsoft Excel 2016. Statistical analysis was performed with SPSS Statistics 24.0, where one-way analysis of variance (ANOVA) was conducted to generate relevant graphical representations. All original data of the indicators were initially subjected to normality testing using the Shapiro–Wilk test (α = 0.05) and to homogeneity of variance testing using Levene’s test (α = 0.05). The results indicated that the data met the assumptions of normal distribution (*p* > 0.05) and homogeneity of variance (*p* > 0.05), thereby satisfying the prerequisites for the application of ANOVA. The Grubbs test (α = 0.05) was employed to identify potential outliers. Identified outliers were subsequently addressed using the intra-group mean replacement method to minimize their impact on the statistical analysis. For post hoc multiple comparisons, the least significant difference (LSD) method was utilized, and the Bonferroni correction was applied to control for Type I error inflation. Results were expressed as mean ± standard error (SE), with alphabetical labeling indicating statistical significance: distinct lowercase letters denote significant differences (*p* < 0.05), while shared letters indicate no significant difference (*p* > 0.05).

## 3. Results

### 3.1. Growth Performance

As shown in Table 2, compared with the CK group, feeding red claw crayfish diets supplemented with varying levels of *R. mucilaginosa* (HL group: 1 × 10^6^ CFU/g; HM group: 1 × 10^7^ CFU/g; HH group: 1 × 10^8^ CFU/g) for 56 days significantly increased the final weight, WGR, SGR, and HSI (*p* < 0.05). Notably, the HM group exhibited the highest values for final weight, WGR, SGR, and HSI, which were significantly higher than those in the HL group and HH group (*p* < 0.05).

Compared with the CK group, the BLG and SR of red claw crayfish in the HM group and HH group significantly increased (*p* < 0.05). Notably, the HM group exhibited the highest values for BLG and SR, which were significantly higher than those in the HH group (*p* < 0.05). However, the BLG and SR in the HL group were not significantly different from those in the CK group (*p* > 0.05).

Compared with the CK group, the FCR of red claw crayfish in the HL group, HM group, and HH group significantly decreased (*p* < 0.05). Notably, the HM group exhibited the lowest value for FCR, which was significantly lower than that in the HL group and HH group (*p* < 0.05).

### 3.2. Digestive Enzyme Activity

As shown in Table 3, compared with the CK group, feeding red claw crayfish diets supplemented with varying levels of *R. mucilaginosa* (HL group: 1 × 10^6^ CFU/g; HM group: 1 × 10^7^ CFU/g; HH group: 1 × 10^8^ CFU/g) for 56 days significantly increased the activity of lipase in the intestine of red claw crayfish (*p* < 0.05). Notably, the HH group exhibited the highest value for lipase, which was significantly higher than that in the HL group (*p* < 0.05) but not significantly different from the HM group (*p* > 0.05).

Compared with the CK group, the activity of trypsin in the intestine of red claw crayfish in the HL group and HM group significantly increased (*p* < 0.05). However, the trypsin in the HH group was not significantly different from that in the CK group (*p* > 0.05).

The activity of α-amylase in the intestine of red claw crayfish in the HL group, HM group, and HH group was not significantly different from that in the CK group (*p* > 0.05).

Compared with the CK group, the activity of trypsin in the hepatopancreas of red claw crayfish in the HL group, HM group, and HH group significantly increased (*p* < 0.05). Notably, the HM group exhibited the highest value for trypsin, which was significantly higher than that in the HL group (*p* < 0.05) but not significantly different from the HH group (*p* > 0.05).

Compared with the CK group, the activities of lipase and α-amylase in the hepatopancreas of red claw crayfish in the HM group and HH group significantly increased (*p* < 0.05). However, the lipase and α-amylase activities in the HL group were not significantly different from those in the CK group (*p* > 0.05).

### 3.3. Gene Expression

**Figure 1:**
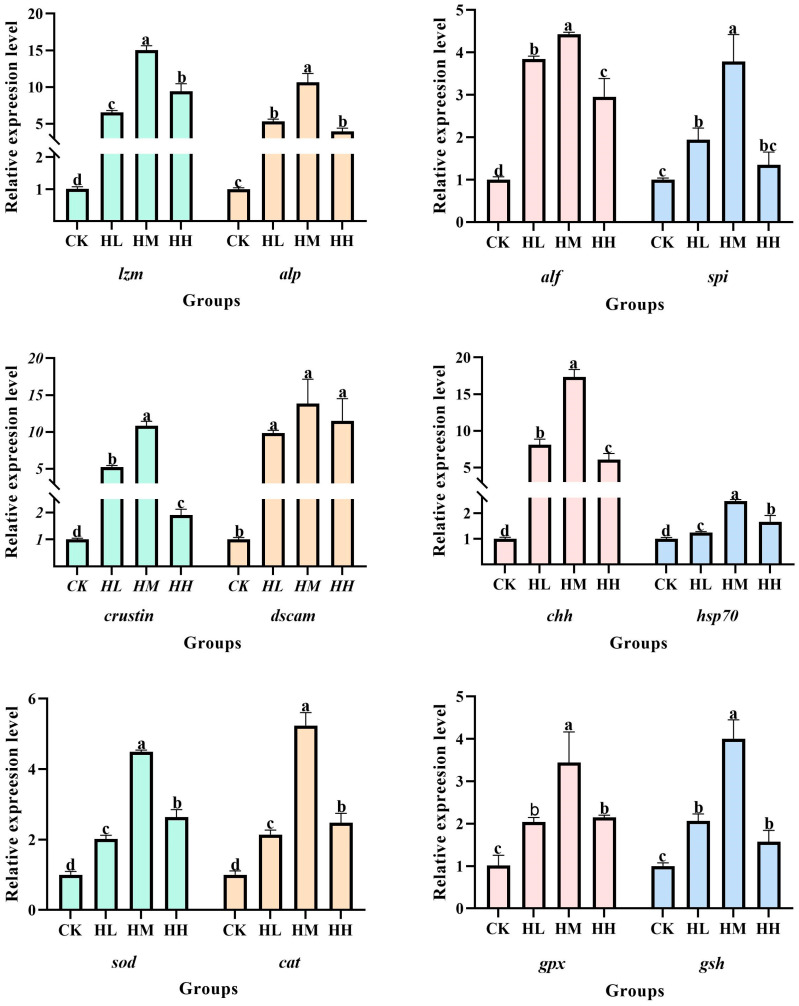
The effect of *R. mucilaginosa* supplementation on immune and antioxidant gene expression in the hepatopancreas of red claw crayfish. All the data are presented as mean ± SE (*n* = 3). According to the LSD test, the mean values marked with different superscript letters in the same figure are significantly different from each other (*p* < 0.05).

As shown in Figure 1, compared with the CK group, feeding red claw crayfish diets supplemented with varying levels of *R. mucilaginosa* (HL group: 1 × 10^6^ CFU/g; HM group: 1 × 10^7^ CFU/g; HH group: 1 × 10^8^ CFU/g) for 56 days significantly upregulated the relative expression levels of *lzm*, *alp*, *alf*, *spi*, *crustin*, *chh*, *hsp70*, *sod*, *cat*, *gpx*, and *gsh* genes in the hepatopancreas of red claw crayfish (*p* < 0.05). Notably, the HM group exhibited the highest values for *lzm*, *alp*, *alf*, *spi*, *crustin*, *chh*, *hsp70*, *sod*, *cat*, *gpx*, and *gsh* genes, which were significantly higher than those in the HL group and HH group (*p* < 0.05).

Compared with the CK group, the relative expression level of the *dscam* gene in the hepatopancreas of red claw crayfish in the HL group, HM group, and HH group was significantly upregulated (*p* < 0.05). However, there was no significant difference in the relative expression level of the *dscam* gene among the HL group, HM group, and HH group (*p* > 0.05).

### 3.4. Gut Microbiota

As shown in Table 4, a total of 1,257,651 valid sequences were generated following the optimization of the original data obtained from sequencing the intestinal samples of red claw crayfish in the CK group, HL group, HM group, and HH group (a total of 12 samples). Among these, 1,059,567 sequences were successfully annotated to the species level.

**Figure 2:**
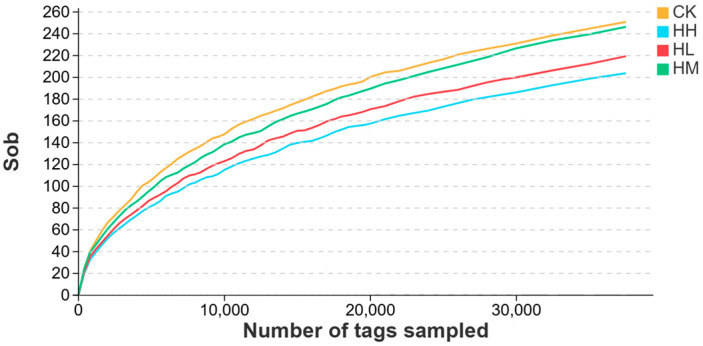
Dilution curves of gut microbiota in red claw crayfish.

As shown in Figure 2, the trend of the dilution curve suggested that, as the number of samples increased, the diversity of bacterial species within the intestinal microbial community of each group exhibited an upward trend.

**Figure 3:**
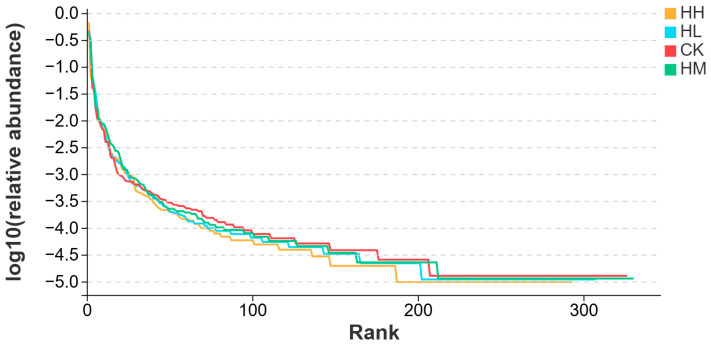
Abundance level curve (OTU level).

As shown in Figure 3, the abundance–grade curve illustrated that the CK group, HL group, HM group, and HH group exhibited an extended span along the horizontal axis and gradually approached a plateau. This suggested that the intestinal microbial species composition within each group was more homogeneous, with higher species richness. Notably, the HM group demonstrated the greatest microbial diversity and the highest level of uniformity.

As shown in Table 5, the high coverage rate of the sequencing results further substantiated that most microbial species within the samples were adequately represented at the current sequencing depth, thus ensuring sufficient data for subsequent bacterial community analysis.

**Figure 4:**
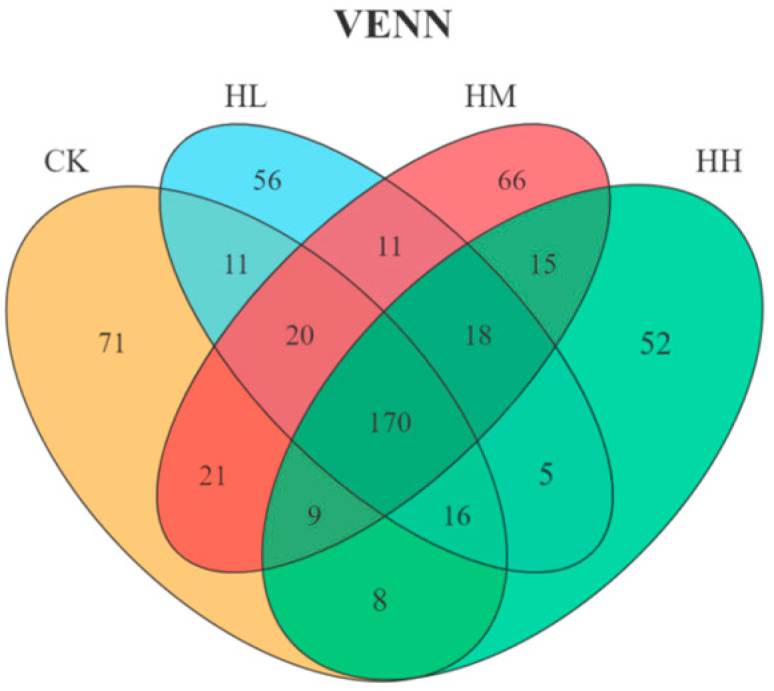
Comparison of OTUs of gut microbiota in red claw crayfish.

**Figure 5:**
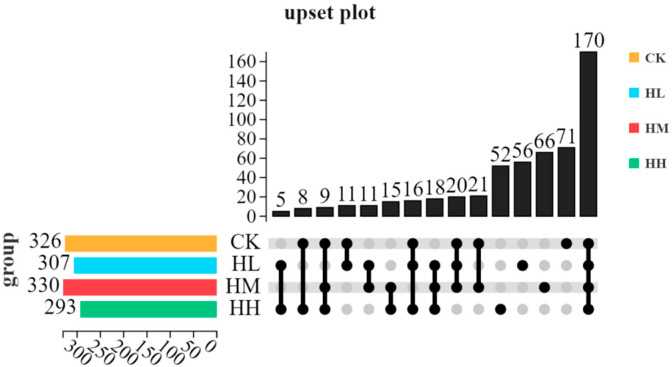
OTUs upset diagram.

As shown in Figure 4 and Figure 5, a total of 549 operational taxonomic units (OTUs) were identified within the intestinal bacteria of red claw crayfish. Specifically, 326 OTUs were detected in the CK group, 307 were detected in the HL group, 330 were detected in the HM group, and 293 were detected in the HH group. Furthermore, the intestinal microbiota of the CK group, HL group, HM group, and HH group contained 71, 56, 66, and 52 OTUs, respectively, which were classified into 17 phyla, 32 classes, 80 orders, 103 families, and 107 genera.

As shown in Table 6, no significant differences were observed in the Shannon index, Simpson index, Chao1 index, and Ace index among the HL group, HM group, and HH group compared to the control group (*p* > 0.05).

**Figure 6:**
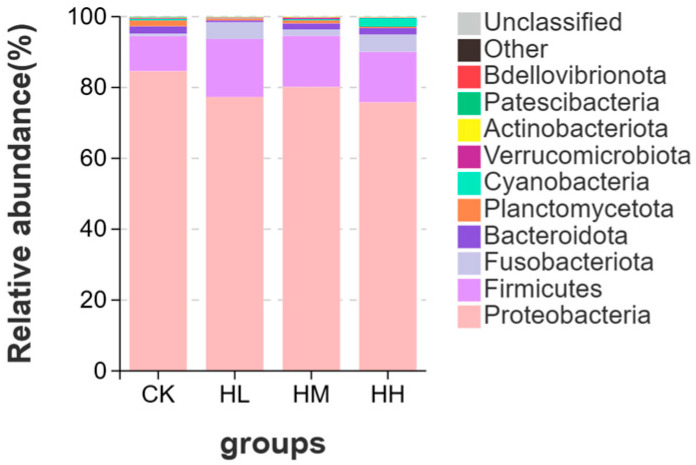
Analysis of relative abundance at the level of gut microbiota.

The composition and relative abundance of the top 10 bacterial communities in intestinal samples from the CK group, HL group, HM group, and HH group at the phylum level are presented in Figure 6. The intestinal microbial communities of the CK group, HL group, HM group, and HH group were predominantly composed of Proteobacteria, Firmicutes, Fusobacteriota, and other bacterial phyla. Differences were observed in the specific abundances of these bacterial communities across the groups. Notably, Proteobacteria constituted the dominant phylum, with relative abundances of 84.58% (CK), 77.31% (HL), 80.10% (HM), and 75.79% (HH). In comparison, the CK group exhibited higher abundances of Proteobacteria, Bacteroidota, and Actinobacteriota compared to the CK group, HL group, HM group, and HH group, whereas the CK group, HL group, HM group, and HH group showed higher abundances of Firmicutes and Fusobacteriota than the CK group. Additionally, the abundance of Verrucomicrobiota in the HM group was significantly higher than that in the CK group (*p* < 0.05).

**Figure 7:**
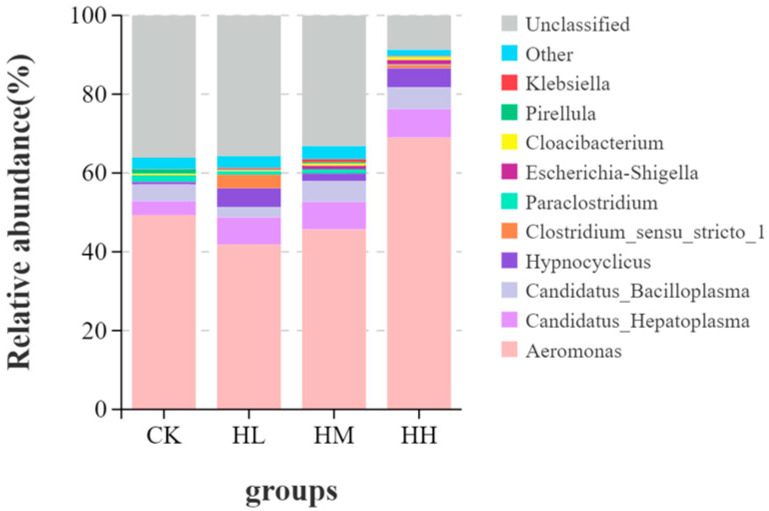
Analysis of the relative abundance of gut microbiota at the genus level.

As shown in Figure 7, by screening for bacterial genera with a relative abundance exceeding 1.00% in the CK group, HL group, HM group, and HH group, it was observed that *Aeromonas*, *Candidatus_Hepatoplasma*, and *Candidatus_Bacilloplasma* were the predominant bacterial species in the intestinal flora of the crayfish. Specifically, the relative abundance of *Aeromonas* in the HH group (71.56%) was significantly higher than that in the CK group (52.27%), HL group (43.91%), and HM group (48.00%) (*p* < 0.05). However, no statistically significant differences were observed among the CK, HL, and HM groups (*p* > 0.05).

**Figure 8:**
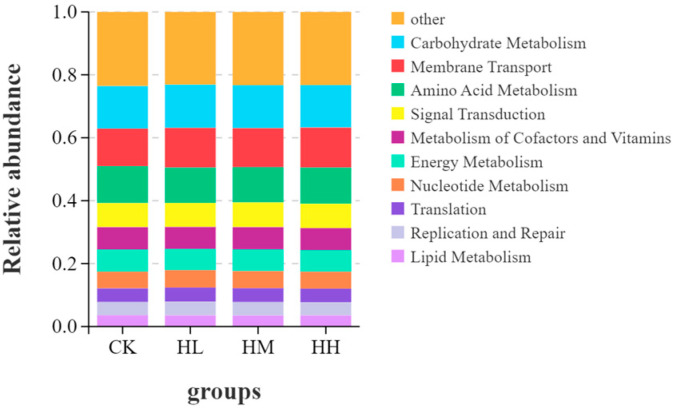
Stacking diagram of gut microbiota function.

**Figure 9:**
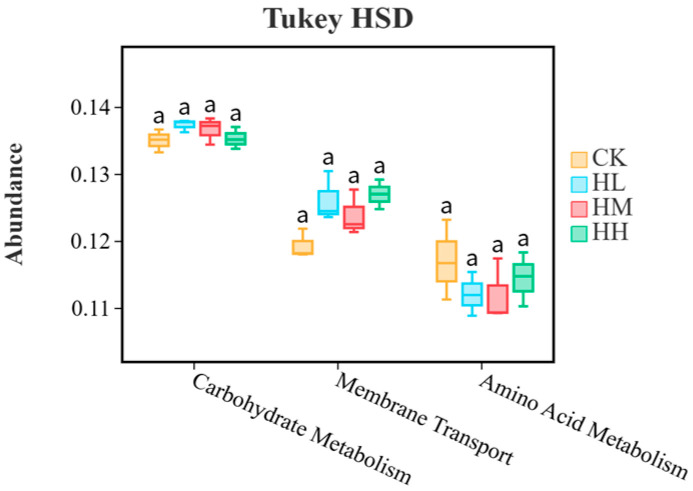
Functional differences of gut microbiota in red claw crayfish. All the data are presented as mean ± SE (*n* = 3). According to the LSD test, the mean values marked with different superscript letters in the same figure are significantly different from each other (*p* < 0.05).

As shown in Figure 8, the relative abundance of KEGG-annotated functional pathways within the intestinal bacterial communities of the CK group, HL group, HM group, and HH group was determined based on KEGG annotation. The enriched KEGG pathways in the intestinal bacterial communities across these four groups were predominantly associated with carbohydrate metabolism, amino acid metabolism, and cofactor/vitamin metabolism. As shown in Figure 9, Tukey’s honestly significant difference (HSD) rank sum test was employed to further analyze the differences among the groups. ANOVA results indicated significant variations in the median functions related to carbohydrate metabolism, membrane transport, and amino acid metabolism between the groups (*p* < 0.05). Post hoc multiple comparisons revealed that, when comparing the treatment groups (HL, HM, HH) with the CK group, no statistically significant differences were observed in the three metabolic functions (*p* > 0.05).

### 3.5. Intestinal Tissue Structure

**Figure 10:**
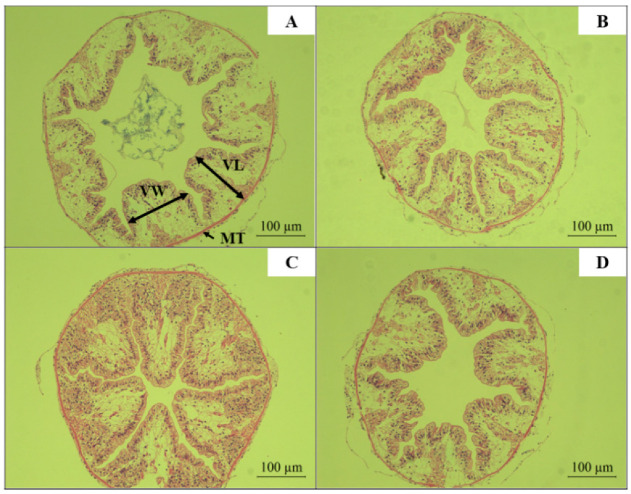
The effect of *R. mucilaginosa* supplementation on the villus length (VL), villus width (VW), and intestinal muscle thickness (MT) of red claw crayfish. (**A**) CK group; (**B**) HL group; (**C**) HM group; (**D**) HH group.

As shown in Table 7 and Figure 10, compared with the CK group, feeding red claw crayfish a diet supplemented with *R. mucilaginosa* (HM group: 1 × 10^7^ CFU/g) for 56 days significantly increased the villus length (VL) and villus width (VW) in the intestine of red claw crayfish (*p* < 0.05). However, there was no significant difference in the VL and VW among the HL group (1 × 10^6^ CFU/g), HH group (1 × 10^8^ CFU/g), and CK group (*p* > 0.05).

Compared with the CK group, the intestinal muscle thickness (MT) of red claw crayfish in the HM group and HH group significantly increased (*p* < 0.05). However, there was no significant difference in the MT between the HL group and the CK group (*p* > 0.05).

## 4. Discussion

The results of this study indicate that feeding diets supplemented with *R. mucilaginosa* significantly promoted increases in WGR, BLG, HSI, SGR, and SR of red claw crayfish while effectively reducing the feed conversion ratio. When the dietary supplementation level of *R. mucilaginosa* reached 1.0 g/kg, the promoting effect on the growth performance of red claw crayfish was most significant. The experimental results demonstrate that *R. mucilaginosa* can actively promote the growth and development of red claw crayfish. This may be attributed to the following: (1) The yeast contains abundant nutrients, including various amino acids, fatty acids, vitamins, minerals, and immunomodulatory compounds, which play important roles in the growth and development of aquatic animals [23], and specifically, the rich content of vitamin E and carotenoids serves as effective nutritional enhancers for animals [24]; (2) yeast can produce abundant oleic acid, an unsaturated fatty acid that plays a crucial role in reducing energy consumption and improving growth and feed efficiency in aquatic animals [25]; (3) the yeast cell wall contains components such as β-glucans and mannan oligosaccharides, whose deposition can also promote body growth, nutrient digestibility, and muscle composition [26]. Similar findings have been reported in other aquatic species. Van Doan et al. observed significantly improved growth performance in Nile tilapia-fed red yeast-supplemented diets for 90 days [27]. Yang et al. reported that Pacific white shrimp (*Litopenaeus vannamei*) fed diets containing marine red yeast for 6 weeks showed significantly enhanced growth performance, whether administered as dried yeast or live yeast [28]. Wang et al. found that supplementation of yeast culture to low-fishmeal diets improves growth, intestinal health, and heat stress resistance in juvenile Chinese mitten crab (*Eriocheir sinensis*) [29].

The experimental results revealed that the growth performance of red claw crayfish in the 10.0 g/kg *R. mucilaginosa* supplementation group was reduced, indicating that excessive addition may adversely affect crayfish growth. This may be related to the rich β-glucan content in *R. mucilaginosa*. Excessive β-glucan present in *R. mucilaginosa* could impair nutrient absorption and digestion in crayfish, leading to reduced growth performance [11].

The experimental results revealed that the intestines and hepatopancreas serve as crucial sources of digestive enzymes, aiding the organism in breaking down nutrients such as proteins, fats, and carbohydrates from food to provide energy for growth and activity [30]. Assessing the activities of protease, lipase, and α-amylase can evaluate the ability of red claw crayfish to digest and absorb nutrients from food in their intestines [31]. This study found that dietary supplementation with varying levels of *R. mucilaginosa* significantly enhanced protease and lipase activities in the intestines of red claw crayfish as well as trypsin, lipase, and α-amylase activities in the hepatopancreas. These results indicate that *R. mucilaginosa* can enhance digestive enzyme activity in red claw crayfish, improving their absorption and digestion efficiency of nutrients in feed, thereby promoting growth and development. The underlying reasons for this phenomenon may include the following: (1) Probiotics can produce digestive enzymes, so when *R. mucilaginosa* is ingested by red claw crayfish, it may generate additional digestive enzymes to facilitate digestion [32]; (2) as probiotics produce digestive enzymes, the organism itself may also produce higher levels of digestive enzymes to adapt to the increased probiotic concentration within the body [33]. Similar findings have been reported. Kuebutornye et al. observed that probiotic supplementation in feed not only supports host nutrition by providing essential nutrients but also improves digestion and nutrient absorption by increasing the activity of digestive enzymes (e.g., amylase, protease, lipase, and cellulase) in the intestines [33]. Wang et al. found that dietary supplementation with red yeast (*R. benthica*) enhanced certain digestive enzyme activities in sea cucumber (*Apostichopus japonicus*) while studying its effects on growth performance and digestive enzyme activity [32].

The experimental results indicate that dietary supplementation with *R. mucilaginosa* significantly upregulated the relative expression levels of *sod*, *gpx*, *gsh*, and *cat* genes in the hepatopancreas of red claw crayfish, demonstrating enhanced antioxidant capacity. This occurs because CAT, GPX, SOD, and GSH constitute the primary antioxidant system in aquatic animals, playing a crucial role in scavenging free radicals and maintaining cellular integrity and organismal health [34]. Specifically, SOD and CAT synergistically eliminate free radicals and reactive oxygen species (ROS): SOD specifically catalyzes the conversion of superoxide anions into oxygen and hydrogen peroxide, while CAT subsequently decomposes hydrogen peroxide into water and oxygen, effectively controlling hydrogen peroxide concentration. This collaborative action maintains cellular physiological processes and reduces damage to cellular structure and function [35]. Similarly, as key components in the antioxidant defense of aquatic animals, GSH enhances cellular resistance to toxic substances, mitigating cellular damage and death during oxidative stress [36], while GPX maintains cellular homeostasis by metabolizing ROS, collectively protecting cells from oxidative damage [37]. Similar studies corroborate these findings. Liu et al. reported that appropriate dietary supplementation with red yeast significantly increased GPX, SOD, and CAT activities in the serum and liver of GIFT tilapia, effectively enhancing antioxidant capacity [20]. Yang et al. observed an upward trend in the relative expression of antioxidant genes (*cat*, *gpx*, *sod*) in Pacific white shrimp after marine red yeast dietary supplementation [28]. Castex et al. found increased overall antioxidant levels in Pacific blue shrimp (*Litopenaeus stylirostris*) fed diets supplemented with the probiotic *Pediococcus acidilactici*, characterized by enhanced activities of SOD, CAT, and GPX [38]. Dietary probiotic supplementation upregulates the expression of antioxidant-related genes, consistent with the findings of Kheirabadi et al. in rainbow trout (*Oncorhynchus mykiss*) [39].

The experimental results revealed that dietary supplementation with *R. mucilaginosa* significantly enhanced the expression levels of immune-related genes in red claw crayfish, including *lzm*, *alf*, *chh*, *spi*, *hsp70*, *alp*, *crustin*, and *dscam*. Crustin, as a core antimicrobial protein in crustacean innate immunity, exhibits upregulated expression that directly strengthens the crayfish’s ability to eliminate pathogens such as bacteria and viruses. Crustin enhances anti-infection defenses in red claw crayfish by disrupting the cell membrane structure of pathogenic cells [40]. Pooljun et al., in a study where whiteleg shrimp (*Penaeus vannamei*) postlarvae were fed diets supplemented with the probiotics Lactobacillus acidophilus and Saccharomyces cerevisiae, observed significant increases in *crustin* gene expression in both hemocytes and the hepatopancreas, accompanied by enhanced protection against bacterial infection [41]. Significantly elevated *dscam* gene expression following marine red yeast supplementation indicates enhanced pathogen resistance in red claw crayfish. This is attributed to Dscam’s ability to specifically recognize and bind pathogen surface molecules (e.g., lipopolysaccharides or peptidoglycans), thereby protecting the crayfish from bacterial invasion by promoting hemocyte phagocytosis and activating downstream immune signaling pathways [42]. The increased expression of *hsp70* in red claw crayfish signifies enhanced immunomodulatory capacity. In crustaceans, HSP70 regulates immune responses not only by stabilizing the conformation of immune cell proteins to maintain immune homeostasis but also by directly inhibiting the proliferation of pathogenic microorganisms (e.g., bacteria or viruses) [43]. This finding aligns with El-Bab et al., who observed yeast additive-induced upregulation of *hsp70* in gilthead seabream (*Sparus aurata*) [44]. LZM, as a broad-spectrum antibacterial effector molecule, plays a vital role in the crustacean immune defense system. LZM directly disrupts pathogen structure by hydrolyzing the peptidoglycan component of their cell walls. Consequently, the increased *lzm* expression in red claw crayfish after marine red yeast supplementation demonstrates that it can enhance broad-spectrum antibacterial capability, expanding the scope of immune defense [45]. Similarly, supplementation with *Bacillus subtilis* and *Bacillus licheniformis* significantly upregulated the relative expression of *hsp70* and *lzm* genes in Nile tilapia (*Oreochromis mossambicus*), enhancing resistance to pathogenic microorganisms and reducing autoimmune diseases [46]. Upregulated expression trends of *chh* and *alp* in red claw crayfish fed marine red yeast-supplemented diets indicate enhancements not only in immunity but also in physiological function. CHH, a multifunctional hormone, participates in molting and growth regulation in crustaceans and indirectly enhances immune responses by activating hemocytes and modulating antioxidant enzyme activity [47]. The upregulation of *alp* gene expression is closely linked to both growth performance and immune defense in red claw crayfish. ALP can attenuate pathogen virulence by hydrolyzing bacterial LPS and also promote skeletal development [48]. This is consistent with Hardy et al., who found that dietary yeast hydrolysate enhanced host immunity in tilapia by upregulating *alp* gene expression [49]. Increased expression of the *spi* gene in red claw crayfish fed marine red yeast indicates an enhanced ability to inhibit pathogen protease activity. SPI protects the host by inhibiting the activity of pathogen proteases, thereby preventing them from invading and disrupting host cell structure and function [50]. Upregulated expression of *alf*, an important immune factor in the hepatopancreas, signifies enhanced resistance to foreign bacteria in red claw crayfish. ALF plays a crucial role in humoral immunity by activating other immune factors within the host to collaboratively defend against pathogenic bacteria [51]. This aligns with Tseng et al., who demonstrated that dietary supplementation with *Lactobacillus plantarum* enhanced immunity and survival rates in white shrimp (*Penaeus vannamei*) [52].

The gut microbiota of aquatic animals plays a pivotal role in host health by modulating nutrient absorption and competing with pathogens [53]. Intestinal microorganisms are not only associated with animal growth [54] but also closely linked to disease phenotypes, such as hepatic disorders and obesity [55]. In this study, high-throughput sequencing of intestinal samples from red claw crayfish fed *R. mucilaginosa*-supplemented diets revealed compositional differences in gut microbiota, indicating that probiotic-enriched feed alters the intestinal microbial communities of farmed species [56]. Gut microbiota is highly susceptible to external factors such as dietary changes and water quality [57], suggesting that *R. mucilaginosa* supplementation likely drives shifts in red claw crayfish intestinal microbial profiles.

Alpha diversity analysis is used to assess the microbial diversity within a single intestinal sample, primarily reflecting two aspects: the number of species (richness) and the uniformity of species distribution (evenness) [58]. In this study, alpha diversity analysis demonstrated no significant differences in microbial evenness, diversity, or richness between the marine red yeast-supplemented groups and the control, implying that *R. mucilaginosa* did not drastically disrupt microbial equilibrium but increased taxonomic variety and abundance within the red claw crayfish gut.

The composition of intestinal microbiota is influenced by multiple factors, such as aquaculture environment, feed composition, probiotic additive dosage, and feeding cycle duration [59]. At the phylum level, Proteobacteria and Bacteroidetes are dominant phyla in the intestinal microbiota of aquatic animals [60]. In crayfish intestines, Proteobacteria is one of the most abundant phyla in the intestinal flora due to its strongest colonization capacity and inclusion of various pathogenic bacteria. A sudden increase in the relative abundance of Proteobacteria in crayfish intestines elevates disease risks in crayfish [61]. Certain pathogenic bacteria within Bacteroidetes can induce multiple microbial diseases in hosts through complex interactions among the host, pathogen, and environment, posing significant threats to aquaculture [62]. The results of this study showed that, in the phylum-level composition of intestinal microbiota in red claw crayfish, the proportions of Proteobacteria and Bacteroidetes were higher in the control group than in the *R. mucilaginosa*-supplemented groups. Notably, Bacteroidetes exhibited an initial decrease followed by an increase. This indicates that dietary *R. mucilaginosa* supplementation inhibits pathogenic bacterial growth, but exceeding a certain additive dosage may disrupt the intestinal microecological environment, leading to the resurgence of opportunistic pathogenic bacteria [63].

The phylum *Verrucomicrobiota* can utilize complex oligosaccharides as fermentation substrates to restore intestinal epithelial barrier function [64]. In this study, the proportion of *Verrucomicrobiota* in the intestinal tract of red claw crayfish in the HM group was higher than that in the control group, indicating that marine red yeast can maintain normal intestinal barrier function. The reason for this phenomenon may be that yeast secretes mannan oligosaccharides (MOS), which competitively block pathogen adhesion to the gastrointestinal mucosa by occupying attachment sites, thereby reducing pathogenic bacterial populations. This creates ecological conditions for the proliferation of beneficial bacteria such as *Verrucomicrobiota*, preserving intestinal epithelial integrity [65]. Gainza et al. supplemented MOS in the diet of Pacific white shrimp and observed lower proportions of Proteobacteria and higher proportions of *Verrucomicrobiota* in the intestinal microbiota of the MOS group compared to the control group [66].

The phylum Firmicutes has been shown to play a significant role in host health through metabolic activities [67]. Firmicutes effectively produce short-chain fatty acids (SCFAs), providing essential nutrients for intestinal mucosal cells and regulating the intestinal microenvironment [68]. Additionally, Firmicutes enhances digestive efficiency and immune function, thereby improving overall host immunity [69]. The results of this study revealed that the proportion of Firmicutes in the intestinal microbiota of red claw crayfish in marine red yeast-supplemented groups was higher than in the control group, indicating that marine red yeast enhances intestinal immune performance and modulates the intestinal microenvironment in red claw crayfish. This may be attributed to bioactive compounds in marine red yeast, such as β-glucans and mannan oligosaccharides (MOS), which stimulate immunity by enhancing resistance to infectious pathogens [10]. Specifically, β-glucans increase antibody concentrations and activate macrophage activity, thereby improving resistance to pathogenic bacteria and reducing their populations [11], creating a favorable environment for the proliferation of beneficial bacteria such as Firmicutes.

The genus Aeromonas can cause diseases in aquatic animals and may even be transmitted to humans through aquatic animals, posing threats to both farmed animals and human health [70]. In the genus-level composition of intestinal microbiota in red claw crayfish, the proportion of Aeromonas in the intestinal microbial genus composition of control-group crayfish was higher than in the HL and HM marine red yeast groups. However, the proportion of Aeromonas increased in the intestinal microbiota of the HH group crayfish. This indicates that dietary marine red yeast supplementation reduces the proportion of pathogenic bacteria in the host intestine, but the probiotic additive dosage must remain within an appropriate range, as excessive amounts may disrupt the host intestinal microbial composition [71]. In this study, the proportion of Aeromonas in the HH group (10.0 g/kg) was observed to increase, which may be attributed to the following factors. First, an excessive dosage of Saccharomyces cerevisiae may supply additional carbon sources or growth-promoting factors that facilitate the proliferation of Aeromonas. Previous studies have indicated that high concentrations of yeast metabolites, such as certain amino acids, can enhance the growth of opportunistic pathogenic bacteria [63]. Furthermore, functional prediction of the gut microbiota in the HH group revealed elevated activity in amino acid metabolic pathways, potentially creating a favorable environment for Aeromonas proliferation. Second, while a moderate amount of Myxyeasts may inhibit pathogenic bacteria through competition for colonization sites—as evidenced by the HM group, where beneficial bacteria such as Verrucomicrobiota were increased—excessive doses may overstimulate the intestinal immune response, leading to dysbiosis of the microbial community [69]. This disruption may subsequently provide an ecological niche for the dominant colonization of Aeromonas.

The intestine is a critical component of the digestive system, and its morphology and structure are essential for nutrient absorption and maintenance of normal intestinal function [71]. The length and width of the intestine determine its surface area, which is a key factor for nutrient absorption. Longer and wider intestines provide a larger surface area, facilitating efficient nutrient absorption [72]. The results of this study found that dietary supplementation with *R. mucilaginosa* significantly increased the intestinal villus length and width of red claw crayfish. Similar findings were reported by Stephen et al., who demonstrated that diets supplemented with red yeast significantly improved intestinal villus length and width in Pacific white shrimp, promoting their growth. Yuan et al. revealed that feeding yeast hydrolysate significantly increased intestinal villus length in Jian carp (*Cyprinus carpio var. Jian*) juveniles and enhanced fish growth performance [73].

## 5. Conclusions

This study demonstrated that feeding red claw crayfish with diets supplemented with varying levels of *R. mucilaginosa* enhanced the growth performance, digestive enzyme activities, proportion of beneficial bacteria, and intestinal villus length and width and upregulated the expression levels of immune and antioxidant-related genes. The optimal marine red yeast supplementation level was determined to be 1.0 g/kg. These findings provide a scientific basis for the development of functional feeds aimed at enhancing productivity and health in sustainable aquaculture practices.

## Figures and Tables

**Table 1:** Primer sequences for RT-PCR of genes in the red claw crayfish.

Gene	Primer Sequence (5′→3′)	Amplicon Size (bp)	Tm (°C)	Gene Bank
*β-actin* ^1^	F: ATCACTGCTCTGGCTCCTGCTACC	148	60	XM_053799524.1
	R: CGGACTCGTCGTACTCCTCCTTGG			
*sod* ^2^	F: GACTACACGGCTTCCACGTC	122	60	XM_053774464.1
	R: AGCGTGGCGTTCTAAGTCAT			
*hsp70* ^3^	F: AGAAGGCAGGCACAGGAGACTC	91	60	XM_053796258.1
	R: TCAATGCTCAGGACAGACACATCG			
*dscam* ^4^	F: CCGAAGTGGCTGGATCTCAATGTC	104	60	XM_053770654.1
	R: TTCTTGCGACGAGTAACTGCTACAC			
*crustin* ^5^	F: CTATGGTAGTGGTGGTTGTGGTTGG	145	60	XM_053794211.1
	R: GGAAGCAGTGGAACGTGAGGTG			
*gpx* ^6^	F: CAAGGTGGTGTTGGTGGTCA	228	60	XM_053777058.1
	R: TCAAAGAAGGTCATCAGAGGCT			
*gsh* ^7^	F: CCCACAAACTGGACCTCCTTCAAG	138	60	XM_053799781.1
	R: CGGCTGCTTCAATGGTAGACACTG			
*cat* ^8^	F: GACCGTCAGCATCTTGTGGAGAAC	94	60	XM_053771460.1
	R: AAGCCTGGGTGAAATTGCCGATAG			
*alf* ^9^	F: GAAGCAGGTCTGGTGTCATTGGAG	91	60	XM_053782181.1
	R: TGCTGGGCTGATTGTTCTGTGATG			
*chh* ^10^	F: TCTCCGCTCAGCCCTCTACAATG	91	60	XM_053788088.1
	R: AGTGAACGCCCAGAACACACCG			
*spi* ^11^	F: TGGTCAACGCAGCTTACTTCAAGG	147	60	XM_053782018.1
	R: TGGAGTGGACTAGCCTGAGATTGG			
*lzm* ^12^	F: TGGTCAACGCAGCTTACTTCAAGG	190	60	XM_053797520.1
	R: TGGAGTGGACTAGCCTGAGATTGG			
*alp* ^13^	F: GCAACAGGAACGGTAGCAAG	190	60	XM_053797520.2
	R: GCCACCCAAGCGGAATAT			

Note: F: Forward primer. R: Reverse primer. ^1^ *β-actin*: Non-regulated reference gene. ^2^ *sod*: Superoxide dismutase. ^3^ *hsp70*: Heat shock protein 70. ^4^ *dscam*: Down syndrome cell adhesion molecule. ^5^ *crustin*: Crustacean antimicrobial peptide. ^6^ *gpx*: Glutathione peroxidase. ^7^ *gsh*: Glutathione. ^8^ *cat*: Catalase. ^9^ *alf*: Anti-lipopolysaccharide factor. ^10^ *chh*: Crustacean hyperglycemic hormone. ^11^ *spi*: Serine protease inhibitor. ^12^ *lzm*: Lysozyme. ^13^ *alp*: Alkaline phosphatase.

**Table 2:** The effect of *R. mucilaginosa* supplementation on the growth performance of red claw crayfish.

Index	Groups			
	CK	HL	HM	HH
Initial weight (g)	0.10 ± 0.00	0.10 ± 0.00	0.10 ± 0.00	0.10 ± 0.00
Final weight (g)	7.77 ± 0.09 ^d^	8.37 ± 0.23 ^c^	11.10 ± 0.10 ^a^	9.67 ± 0.18 ^b^
WGR ^1^ (%)	7440.45 ± 85.62 ^d^	8022.98 ± 226.54 ^c^	10,676.7 ± 97.09 ^a^	9285.11 ± 171.25 ^b^
BLG ^2^ (%)	940.4 ± 10.01 ^c^	963.52 ± 15.29 ^bc^	1032.88 ± 15.78 ^a^	998.2 ± 25.19 ^b^
SGR ^3^ (%/day)	6.18 ± 0.02 ^d^	6.28 ± 0.04 ^c^	6.69 ± 0.01 ^a^	6.49 ± 0.03 ^b^
HIS ^4^ (%)	7.30 ± 0.06 ^c^	7.81 ± 0.03 ^b^	8.17 ± 0.18 ^a^	7.79 ± 0.05 ^b^
FCR ^5^	1.27 ± 0.01 ^a^	1.23 ± 0.01 ^b^	1.18 ± 0.01 ^c^	1.21 ± 0.01 ^b^
SR ^6^ (%)	88.89 ± 0.56 ^c^	91.11 ± 0.56 ^bc^	95.00 ± 0.96 ^a^	91.67 ± 0.96 ^b^

Note: All the data are presented as mean ± SE (*n* = 3). According to the LSD test, the mean values marked with different superscript letters in the same row are significantly different from each other (*p* < 0.05). ^1^ WGR: Weight gain rate. ^2^ BLG: Body length growth rate. ^3^ SGR: Specific growth rate. ^4^ HSI: Hepatosomatic index. ^5^ FCR: Feed conversion rate. ^6^ SR: Survival rate.

**Table 3:** The effect of *R. mucilaginosa* supplementation on the digestive enzyme activity of red claw crayfish.

Index	Groups			
	CK	HL	HM	HH
Intestine				
Trypsin (U/mg prot)	1151.99 ± 57.25 ^b^	1436.19 ± 88.15 ^a^	1540.72 ± 74.72 ^a^	1192.69 ± 63.19 ^b^
Lipase (U/g prot)	15.34 ± 0.74 ^c^	19.17 ± 0.57 ^b^	20.82 ± 0.75 ^ab^	22.27 ± 0.90 ^a^
α-amylase (U/mg prot)	0.34 ± 0.00	0.35 ± 0.00	0.34 ± 0.00	0.35 ± 0.00
Hepatopancreas				
Trypsin (U/mg prot)	896.51 ± 67.18 ^c^	1124.09 ± 112.71 ^b^	1399.63 ± 93.93 ^a^	1203.19 ± 70.76 ^ab^
Lipase (U/g prot)	14.37 ± 0.57 ^c^	15.70 ± 0.71 ^bc^	18.30 ± 0.99 ^a^	17.29 ± 0.72 ^ab^
α-amylase (U/mg prot)	0.26 ± 0.02 ^b^	0.29 ± 0.00 ^ab^	0.33 ± 0.01 ^a^	0.30 ± 0.02 ^a^

Note: All the data are presented as mean ± SE (*n* = 3). According to the LSD test, the mean values marked with different superscript letters in the same row are significantly different from each other (*p* < 0.05).

**Table 4:** Statistics of tag and OTU quantity.

Sample	Total Tags	Taxon Tags	Singleton Tags	Operational Taxonomic Units (OTUs)
CK-1	49,308	39,991	9317	264
CK-2	111,601	94,525	17,076	285
CK-3	113,865	95,915	17,948	353
H-L-1	106,037	88,077	17,960	296
H-L-2	108,810	92,918	15,892	257
H-L-3	107,984	88,009	19,974	301
H-M-1	110,398	89,694	20,704	364
H-M-2	105,628	89,470	16,148	241
H-M-3	98,692	80,121	18,571	349
H-H-1	114,732	99,874	14,858	307
H-H-2	113,643	96,896	16,747	227
H-H-3	116,953	104,077	12,876	298

**Table 5:** Analysis of the coverage rate of gut bacteria in red claw crayfish.

Sample	Good’s Coverage (%)
CK	0.99 ± 0.00
HL	0.99 ± 0.00
HM	0.99 ± 0.00
HH	0.99 ± 0.00

Note: All the data are presented as mean ± SE (*n* = 3).

**Table 6:** Alpha diversity index of gut microbiota in red claw crayfish.

Index	Groups			
	CK	HL	HM	HH
Shannon	2.08 ± 0.17	2.28 ± 0.14	2.40 ± 0.07	1.93 ± 0.21
Simpson	0.54 ± 0.04	0.64 ± 0.02	0.65 ± 0.02	0.50 ± 0.06
Chao1	331.40 ± 27.05	328.38 ± 7.32	365.53 ± 40.05	315.03 ± 26.15
Ace	343.37 ± 24.36	340.54 ± 2.65	375.96 ± 44.83	327.04 ± 26.81

Note: All the data are presented as mean ± SE (*n* = 3).

**Table 7:** The effect of *R. mucilaginosa* supplementation on the intestinal tissue structure of red claw crayfish.

Index	Groups			
	CK	HL	HM	HH
VL ^1^ (μm)	144.52 ± 3.77 ^b^	169.78 ± 1.46 ^ab^	193.64 ± 17.73 ^a^	153.32 ± 2.27 ^b^
VW ^2^ (μm)	130.18 ± 4.96 ^b^	142.74 ± 9.03 ^ab^	152.32 ± 6.72 ^a^	142.11 ± 3.20 ^ab^
MT ^3^ (μm)	11.37 ± 0.51 ^b^	11.33 ± 0.35 ^b^	19.38 ± 0.92 ^a^	12.79 ± 1.92 ^a^

Note: All the data are presented as mean ± SE (*n* = 3). According to the LSD test, the mean values marked with different superscript letters in the same row are significantly different from each other (*p* < 0.05). ^1^ VL: Villus length. ^2^ VW: Villus width. ^3^ MT: Intestinal muscle thickness.

## Data Availability

The original contributions presented in this study are included in the article, and further inquiries can be directed to the corresponding author(s).
